# Fruit and vegetable consumption among Brazilian adults: trends from 2008 to 2023

**DOI:** 10.1590/0102-311XEN032424

**Published:** 2025-02-07

**Authors:** Izabella Paula Araújo Veiga, Thais Cristina Marquezine Caldeira, Marcela Mello Soares, Taciana Maia de Sousa, Luiza Eunice Sá da Silva, Rafael Moreira Claro

**Affiliations:** 1 Universidade Federal de Minas Gerais, Belo Horizonte, Brasil.; 2 Faculdade de Saúde Pública, Universidade de São Paulo, São Paulo, Brasil.

**Keywords:** Food Consumption, Fruit, Vegetables, Health Surveys, Interrupted Time Series Analysis, Consumo de Alimentos, Frutas, Verduras, Inquéritos Epidemiológicos, Análise de Séries Temporais Interrompida, Consumo de Alimentos, Frutas, Verduras, Encuestas Epidemiológicas, Análisis de Series de Tiempo Interrumpido

## Abstract

Adequate fruit and vegetable consumption is recognized for its health benefits, including preventing noncommunicable diseases, therefore it should be monitored over the years. This study aimed to investigate the temporal trend of fruit and vegetable consumption among Brazilian adults (≥ 18 years) residing in 26 Brazilian capitals and the Federal District from 2008-2023. A time-series analysis of the *Surveillance of Risk and Protective Factors for Chronic Diseases by Telephone Survey* (Vigitel) was conducted. Regular (≥ 5 days/week) and recommended (≥ 5 servings/day on ≥ 5 days/week) fruit and vegetable consumption were analyzed for total population and sociodemographic groups. Prais-Winsten regression models were used to identify trends in the entire (2008-2023), initial (2008-2014) and most recent (2015-2023) periods. Regular and recommended consumption remained stable from 2008 to 2023. From 2008 to 2014, regular consumption increased in total population (0.71pp/year) and all sociodemographic groups, except for adults aged 25-34 years. From 2015 to 2023, regular consumption decreased in the total population (-0.56pp/year), mainly among men (-0.70pp/year), adults aged 25-34 years (-0.84pp/year), and with higher schooling (-0.96pp/year). The recommended consumption increased from 2008 to 2014 (0.81pp/year), mainly among women (0.90pp/year), adults aged 55-64 years (0.96pp/year), and those with higher schooling (0.77pp/year). The recommended consumption decreased from 2015 to 2023 in total population (-0.52pp/year) and all sociodemographic groups, except for men and adults aged ≥ 65 years. The Brazilian fruit and vegetable consumption increased from 2008 to 2014 but reduced recently (2015-2023).

## Abstract (pt)

O consumo adequado de frutas e hortaliças é reconhecido pelos benefícios à saúde, incluindo a prevenção de doenças não transmissíveis; portanto, é importante monitorar esse consumo ao longo dos anos. Este estudo teve como objetivo investigar a tendência temporal do consumo de frutas e hortaliças entre adultos brasileiros (≥ 18 anos) residentes em 26 capitais brasileiras e no Distrito Federal de 2008 a 2023. Foi realizada uma análise de séries temporais do *Sistema de Vigilância de Fatores de Risco e Proteção para Doenças Crônicas por Inquérito Telefônico* (Vigitel). O consumo regular (≥ 5 dias/semana) e recomendado (≥ 5 porções/dia em ≥ 5 dias/semana) de frutas e hortaliças foi analisado para a população total e para grupos sociodemográficos. Modelos de regressão Prais-Winsten foram utilizados para identificar tendências no período total (2008-2023), no período inicial (2008-2014) e no período mais recente (2015-2023). O consumo regular e recomendado manteve-se estável de 2008 a 2023. De 2008 a 2014, o consumo regular aumentou na população total (0,71p.p./ano) e em todos os grupos sociodemográficos, exceto entre adultos de 25 a 34 anos. De 2015 a 2023, o consumo regular diminuiu na população total (-0,56p.p./ano), principalmente entre homens (-0,70p.p./ano), adultos de 25 a 34 anos (-0,84p.p./ano) e aqueles com maior nível de escolaridade (-0,96p.p./ano). O consumo recomendado aumentou de 2008 a 2014 (0,81p.p./ano), principalmente entre mulheres (0,90p.p./ano), adultos de 55 a 64 anos (0,96p.p./ano) e aqueles com maior nível de escolaridade (0,77p.p./ano). O consumo recomendado diminuiu de 2015 a 2023 na população total (-0,52p.p./ano) e em todos os grupos sociodemográficos, exceto entre homens e adultos ≥ 65 anos. O consumo brasileiro de frutas e hortaliças aumentou de 2008 a 2014, mas reduziu recentemente (2015-2023).

## Abstract (es)

El consumo adecuado de frutas y verduras tiene beneficios para la salud, incluido la prevención de enfermedades no transmisibles, lo que hace importante monitorear este consumo a lo largo de los años. Este estudio tuvo como objetivo investigar la tendencia temporal del consumo de frutas y verduras entre adultos brasileños (≥ 18 años) que viven en 26 capitales brasileñas y el Distrito Federal de 2008 a 2023. Se realizó un análisis de series temporales del *Vigilancia de Factores de Riesgo y Protección para Enfermedades Crónicas No Transmisibles por Entrevista Telefónica* (Vigitel). Se analizó el consumo regular (≥ 5 días/semana) y el recomendado (≥ 5 porciones/día en ≥ 5 días/semana) de frutas y verduras para la población total y para los grupos sociodemográficos. Los modelos de regresión Prais-Winsten se utilizaron para identificar las tendencias en el período total (2008-2023), en el período inicial (2008-2014) y en el período más reciente (2015-2023). El consumo regular y el recomendado se mantuvo estable de 2008 a 2023. De 2008 a 2014, el consumo regular aumentó en la población total (0,71pp/año) y en todos los grupos sociodemográficos, excepto entre los adultos de 25 a 34 años. De 2015 a 2023, el consumo regular disminuyó en la población total (-0,56pp/año), principalmente entre hombres (-0,70pp/año), adultos de entre 25 y 34 años (-0,84pp/año) y aquellos con un mayor nivel de educación (-0,96pp/año). El consumo recomendado aumentó de 2008 a 2014 (0,81pp/año), principalmente entre mujeres (0,90pp/año), adultos de entre 55 y 64 años (0,96pp/año) y aquellos con un mayor nivel de educación (0,77pp/año). El consumo recomendado disminuyó de 2015 a 2023 en la población total (-0,52pp/año) y en todos los grupos sociodemográficos, excepto entre hombres y adultos ≥ 65 años. El consumo brasileño de frutas y verduras aumentó de 2008 a 2014, pero disminuyó recientemente (2015-2023).

## Introduction

Adequate fruits and vegetable consumption has an important protective effect on health ^1^ ^,^ ^2^. This benefit stems from their rich micronutrients, vitamins, minerals, fibers, and phytochemicals content, in addition to their low energy density ^2^ ^,^ ^3^. A consistent relation has been established between consuming enough fruit and vegetable and the prevention of noncommunicable diseases (NCD) such as cardiovascular diseases, different types of cancer, diabetes mellitus, and metabolic syndrome ^3^ ^,^ ^4^. The World Health Organization (WHO) recommends a minimum intake of five servings or 400g of fruit and vegetable per day ^5^.

Specifically in Brazil, the *Dietary Guidelines for the Brazilian Population*, proposed by the Brazilian Ministry of Health, aligns with the WHO recommendation by advocating for a diet based on natural and minimally processed foods rather than ultra-processed foods ^1^. It represents a dietary intake made up of a wide diversity of plant- and animal-based foods that have undergone little or no change, such as grains, roots, vegetables, fruits, eggs and fresh meat ^1^.

Despite the long-standing understanding of the benefits of fruit and vegetable consumption, several studies have reported a low prevalence of individuals meeting the recommendation, both in high-income countries and in low- and middle-income countries ^6^. A cross-sectional study showed that 80% of individuals residing in low- and middle-income countries fail to meet adequate amounts of fruit and vegetable as recommended by WHO guidelines ^6^. This finding is concerning as these countries are at risk of being disproportionately affected by NCD in the coming years ^7^.

In Brazil, between 2008 and 2016, there was an increase in the recommended consumption of fruit and vegetable with approximately 24.4% of the adult population meeting adequate consumption of these foods in 2016 ^8^. Although only a quarter of the population reached sufficient fruit and vegetable consumption, this proportion is higher than high-income countries such as the United States, in which only 9% of the population reached the recommendation in 2015 ^9^. This scenario results from efforts by the Brazilian government to promote healthy eating habits ^10^, including fruit and vegetable consumption, as well as intensified actions targeting food and nutrition in primary health care (PHC) during this period ^11^. Furthermore, intersectoral strategies contributed to the economic growth of the country, leading to higher family income ^12^ and more favorable food prices ^10^, both of which are potential determinants of fruit and vegetable consumption.

However, a series of political and economic changes experienced in the country since 2015 ^13^ may have negatively influenced the food consumption patterns of the Brazilian population. Additionally, the COVID-19 pandemic exacerbated the situation by introducing a health emergency and intensifying economic challenges ^14^. In this context, it becomes imperative to continue monitoring the temporal evolution of fruit and vegetable consumption to assess and strengthen public strategies and policies aimed at promoting the consumption of these foods. Thus, this study aimed to analyze the temporal trend of fruit and vegetable consumption among adults in Brazilian capitals and the Federal District from 2008 to 2023, comparing estimates from the initial period (2008-2014) and the most recent period (2015-2023).

## Methods

### Design

This is a time series study based on data collected by the *Surveillance of Risk and Protective Factors for Chronic Diseases by Telephone Survey* (Vigitel, acronym in Portuguese) from 2008 to 2023. Vigitel is a population-based health survey conducted annually with a probabilistic sample of adults (≥ 18 years) living in households in the 26 capitals and the Federal District, provided they have at least one telephone landline (n = 697,549) ^15^ ^,^ ^16^.

### Sample

The sampling process is conducted in two steps. The first step consists of a systematic gathering of 10,000 telephone lines (including landlines and mobile phones in 2023) in each city and identifying the eligible ones. For landline interviews, the second step involves selecting one adult resident from each household (via simple random sampling) to participate in the interview. In the case of mobile phones, the interview is conducted directly with the line user, provided they are adults (≥ 18 years old) ^15^ ^,^ ^16^.

Until 2019, around 2,000 interviews were conducted annually in each city, which allowed an estimation of indicator prevalence with a maximum error of two percentage points and a 95% confidence interval (95%CI) ^15^. During the COVID-19 pandemic in 2020 and 2021, a minimum sample size of 1,000 individuals in each city was established. This adjustment enabled estimating indicator prevalence with a maximum error of four percentage points and a 95%CI ^15^ ^,^ ^16^. However, due to issues in the bidding process of the survey, data collection was not possible in 2022, requiring a simplified operation in 2023. Consequently, further reduction in the sample size was necessary, with a minimum of 800 interviews per city. Despite the reduction, estimates with 95%CI and a maximum error of four percentage points were still achievable ^15^ ^,^ ^16^.

Post-stratification factors are employed to correct the estimates, considering the probability of unequal selection of respondents, adjusting the sociodemographic distribution (sex, age, and schooling) of the sample to the distribution of the adult population of each city. More details about the sampling and weighting processes are provided in the annual reports ^15^ ^,^ ^16^.

### Measures

For this study, the indicators of regular and recommended fruit and vegetable consumption were analyzed. The construction of the variable was based on the fruit and vegetable consumption indicator from the main Brazilian health surveys ^15^ ^,^ ^16^ ^,^ ^17^. Regular fruit and vegetable consumption was estimated based on responses to the following questions: “How many days per week do you usually eat at least one type of vegetable (lettuce, tomato, cabbage, carrot, chayote, eggplant, zucchini - not including potatoes, cassava, or yams)?”; “How many days per week do you usually drink fresh fruit juice?”; and “How many days per week do you usually eat fruit?”. The response options for each of these questions ranged from: 1-2, 3-4, 5-6, every day, rarely, never ^15^ ^,^ ^16^ ^,^ ^17^. Regular consumption was considered in cases in which fruits (including fresh juices) and vegetables (regardless of type) were consumed on five or more days of the week (≥ 5 days/week). It is necessary for individuals to consume both regularly, but the number of servings throughout the day is not considered.

The recommended fruit and vegetable consumption was estimated based on the responses to the following questions: “How many days of the week do you usually eat lettuce and tomato salad or any other raw vegetable salad?” (1-2, 3-4, 5-6, every day, rarely, never); “On an ordinary day, do you eat this type of salad” (at lunch, at dinner, both); “How many days of the week do you usually eat cooked vegetables in a meal or soup, for example, kale, carrot, chayote, eggplant, zucchini - not including potatoes, cassava or yam? (1-2, 3-4, 5-6, every day, rarely, never)”; “On an ordinary day, do you eat cooked vegetables” (at lunch, at dinner, both); “On an ordinary day, how many glasses do you drink of fresh fruit juice?” (1, 2, 3 or more); and “On an average day, how many times do you eat fruit?” (1, 2, 3 or more) ^15^. Regarding fruit consumption, up to three daily servings were considered, with fruit juice consumption counting as one serving. Regarding the consumption of raw and cooked vegetables at lunch and/or dinner, up to four daily servings were considered. Recommended fruit and vegetable intake was achieved when the total servings consumed daily reached at least five servings on at least five days of the week (based on the regular fruit and vegetable consumption indicator, ≥ 5 servings/day on ≥ 5 days/week), following WHO ^5^ and Brazilian ^1^ dietary guidelines.

Sociodemographic variables such as sex (male, female), age group in years (18-24, 25-34, 35-44, 45-54, 55-64, ≥ 65) and schooling level in years of study (0-8, 9-11, ≥ 12) complement the analysis.

### Analysis

The population was described according to sociodemographic characteristics. The prevalence of recommended and regular fruit and vegetable consumption was estimated for the entire population and sociodemographic groups across all years studied. The temporal trend of the indicators was estimated using linear regression models for the entire period (2008-2023), as well as for the initial (2008-2014) and most recent (2015-2023) periods. To assess the impact of political and economic changes in the country from 2015 onwards, the continuous fractional time series was analyzed in this study. From 2015, other studies have noted negative changes in the prevalence of risk factors ^16^, identifying 2015 as a crucial cutoff point for our analysis. By conducting a comparative analysis of periods before and after that year, we aimed to better identify changes in indicators, as significant changes might be masked when analyzing the complete period. Linear regression models - preferably Prais-Winsten or simple linear regression when convergence was not achieved - were used to identify temporal trends for each indicator in the studied period. Prais-Winsten models are used to correct the effect of serial autocorrelation and are recommended in time trend studies ^18^. A positive regression coefficient showed an increase in the indicators in percentual points per year (pp/year), while a negative coefficient indicated a decrease. Significant variation values were considered, p-values less than 0.05.

Stata software version 16.1 (https://www.stata.com) was used to organize and analyze the data. Vigitel databases are available for public access (http://svs.aids.gov.br/download/Vigitel/) and the survey was approved by the Brazilian National Ethics Committee for Research with Human Beings of the Brazilian Ministry of Health.

## Results

Vigitel conducted 697,549 interviews with adult individuals (≥ 18 years) from 2008 to 2023. Over the entire period, a decrease was observed in the proportion of young adults (18-24 years), declining from 17.9% to 12.9% (-0.33pp/year), while an increase in the proportion of individuals aged 55-64 years was observed, rising from 10.4% to 13.9% (0.25pp/year). Regarding schooling level, there was a decrease in individuals with 0 to 8 years of schooling, from 43.7% to 25.8% (-1.28pp/year), and an increase in adults with 12 years of schooling or more, from 21.6% to 32.9% (0.78pp/year) (Table 1).

**Table 1:** Distribution of the adult population (≥ 18 years) of Brazilian capitals and the Federal District according to sociodemographic characteristics. *Surveillance of Risk and Protective Factors for Chronic Diseases by Telephone Survey* (Vigitel), 2008- 2023 (n = 697,549).

Characteristics	2008 (%)	2014 (%)	2015 (%)	2023 (%)	Average increment (2008-2014) (pp/year) *	Average increment (2015-2023) (pp/year) *	Average increment (2008-2023) (pp/year) *
Sex							
Male	46.1	46.1	46.0	46.1	-0.01 **	-0.01 **	-0.02 **
Female	53.9	53.9	54.0	53.9	0.01 **	0.01 **	0.02 **
Age (years)							
18-24	17.9	15.6	15.2	12.9	-0.36 **	-0.39 **	-0.33 **
25-34	25.4	25.3	25.2	24.9	-0.04 **	-0.03 ***	-0.05 **
35-44	20.4	19.6	19.4	18.3	-0.14 **	-0.13 **	-0.15 **
45-54	16.1	17.1	17.3	18.3	0.16 **	0.17 **	0.15 **
55-64	10.4	11.8	12.1	13.9	0.24 **	0.24 **	0.25 **
65 and more	9.8	10.6	10.8	11.8	0.14 **	0.14 **	0.14 **
Schooling (years of study)							
0-8	43.7	35.9	34.6	25.8	-1.30 **	-1.33 **	-1.28 **
9-11	34.7	38.1	38.1	41.3	0.30 **	0.59 ***	0.51 ***
12 and more	21.6	25.9	27.3	32.9	1.00 **	0.78 **	0.78 ***

* Corresponding to the Prais-Winsten regression coefficient of the indicator value over the survey year;

** p < 0.000;

*** p between 0.05-0.001.

The prevalence of regular fruit and vegetable consumption (≥ 5 days/week) remained stable from 2008 to 2023, with an average of 34.1% for the entire population and among all sociodemographic groups. In the initial period (2008-2014), regular fruit and vegetable consumption increased from 33% to 36.5% (0.71pp/year) for the entire population and almost all sociodemographic groups, except for adults aged 25 to 34 years. The highest increase during this period was observed among women (0.75pp/year), individuals aged 65 and more (0.83pp/year), and adults with 0 to 8 years of schooling (0.69pp/year). Conversely, the analysis of the most recent period (2015-2023) showed a decrease in the prevalence of fruit and vegetable consumption for the entire population, from 37.6% to 31.9% (-0.56pp/year), particularly among men (-0.70pp/year), individuals aged 25-34 years (-0.84pp/year) and with more than 12 years of schooling (-0.96pp/year) (Table 2 and Supplementary Material - Table S1: https://cadernos.ensp.fiocruz.br/static//arquivo/suppl-e00032424_6302.pdf).

**Table 2:** Percentage of the adult population (≥ 18 years) that consumes fruits and vegetables on five or more days of the week (regular consumption) in Brazilian state capitals and in the Federal District, according to sociodemographic characteristics. *Surveillance of Risk and Protective Factors for Chronic Diseases by Telephone Survey* (Vigitel), 2008- 2023 (n = 697,549).

Characteristics	2008 (%)	2014 (%)	2015 (%)	2023 (%)	Average increment (2008-2014) (pp/year) *	Average increment (2015-2023) (pp/year) *	Average increment (2008-2023) (pp/year) *
Sex							
Male	26.4	29.4	31.3	27.9	0.67 **	-0.70 **	0.02
Female	38.6	42.5	43.1	35.3	0.75 **	-0.47 **	0.16
Age (years)							
18-24	24.6	27.5	29.3	26.0	0.44 **	-0.66 **	0.10
25-34	29.6	33.9	35.3	28.0	0.78	-0.84 **	0.15
35-44	31.7	33.9	35.7	27.5	0.50 **	-0.61 **	-0.02
45-54	37.0	38.7	39.2	34.0	0.75 **	-0.64 **	-0.05
55-64	40.7	44.6	44.6	37.4	0.76 **	-0.65	-0.07
65 and more	45.3	47.6	48.1	43.5	0.83 **	-0.35	-0.06
Schooling (years of study)							
0-8	29.5	32.4	33.0	28.1	0.69 **	-0.09	0.19
9-11	31.0	33.4	33.8	28.6	0.55 **	-0.89 ***	-0.19
12 and more	43.2	46.5	48.9	38.9	0.58 **	-0.96 **	-0.11
Total	33.0	36.5	37.6	31.9	0.71 **	-0.56 **	0.10

* Corresponding to the Prais-Winsten regression coefficient of the indicator value over the survey year;

** p between 0.05-0.001;

*** p < 0.000.

Recommended fruit and vegetable consumption (≥ 5 servings/day on ≥ 5 days/week) also remained stable from 2008 to 2023, with an average of 22.5% for the entire population and across all sociodemographic groups. In the initial period (2008-2014), recommended fruit and vegetable consumption increased from 20% to 24.1% (0.81pp/year) for the entire population, with almost all sociodemographic groups showing an increase, particularly among women (0.90pp/year), individuals aged 55 to 64 years (0.96pp/year), and those with 12 years or more of schooling (0.77pp/year). The only exception was the group of individuals aged 65 and more, in which no increase was observed. However, from 2015 to 2023, the recommended fruit and vegetable consumption decreased from 25.2% to 21.4% (-0.52pp/year) for almost all sociodemographic groups, except for men, individuals aged 18-24 and aged 65 or more. The largest reductions were observed among women (-0.68pp/year), adults aged 25-34 years (-0.73pp/year) and with 12 years or more of schooling (-0.74pp/year) (Table 3 and Supplementary Material - Table S2: https://cadernos.ensp.fiocruz.br/static//arquivo/suppl-e00032424_6302.pdf).

**Table 3:** Percentage of the adult population (≥ 18 years) that consumes five or more daily servings of fruits and vegetables on five or more days of the week (recommended consumption) in Brazilian state capitals and in the Federal District, according to sociodemographic characteristics. *Surveillance of Risk and Protective Factors for Chronic Diseases by Telephone Survey* (Vigitel), 2008- 2023 (n = 697,549).

Characteristics	2008 (%)	2014 (%)	2015 (%)	2023 (%)	Average increment (2008-2014) (pp/year) *	Average increment (2015-2023) (pp/year) *	Average increment (2008-2023) (pp/year) *
Sex							
Male	15.8	19.3	21.0	19.3	0.71 **	-0.57 ***	0.11
Female	23.7	28.2	28.9	23.2	0.90 ***	-0.48 ***	0.23
Age (years)							
18-24	15.6	19.2	21.0	20.2	0.64 **	-0.63 ***	0.22
25-34	18.3	22.7	25.3	20.8	0.86 ***	-0.76 ***	0.27
35-44	19.4	23.4	24.2	18.2	0.68 ***	-0.40 ***	0.19
45-54	22.3	25.9	26.3	21.5	0.85 ***	-0.49 **	0.04
55-64	23.6	28.7	28.8	24.9	0.96 **	-0.46 ***	0.09
65 and more	26.3	27.8	27.3	24.8	0.57	-0.28	0.04
Schooling (years of study)							
0-8	16.9	20.2	20.1	17.1	0.69 ***	-0.23 **	0.14
9-11	19.6	22.5	23.2	19.5	0.71 ***	-0.79 **	-0.05
12 and more	27.1	31.9	34.6	27.2	0.77 ***	-0.73 ***	0.13
Total	20.0	24.1	25.2	21.4	0.81 ***	-0.52 **	0.17

* Corresponding to the Prais-Winsten regression coefficient of the indicator value over the survey year;

** p < 0.000;

*** p between 0.05-0.001.

## Discussion

From 2008 to 2023, a stability in the prevalence of regular and recommended fruit and vegetable consumption among Brazilian adults was observed. However, when stratifying the period, significant changes in fruit and vegetable consumption were evident, with increased fruit and vegetable consumption in the initial period (2008-2014) followed by a sharp reduction in the most recent period (2015-2023). Among the sociodemographic groups, fruit and vegetable consumption increased in the initial period, especially among women and older adults. There was an increase in regular fruit and vegetable consumption, mainly among adults with lower schooling, and an increase in recommended fruit and vegetable consumption for adults with higher schooling. Conversely, there was a reduction in regular and recommended fruit and vegetable consumption in the most recent period for almost all sociodemographic groups, especially men (regarding regular consumption), individuals aged 25-34 years, and with higher schooling. Overall, the greater prevalence and a larger increase in fruit and vegetable consumption in the initial period among women, older individuals, and those with higher schooling, maintained these groups with the highest prevalence of consumption.

In recent decades, studies have evaluated fruit and vegetable consumption worldwide, including in Brazil. Nationally, population-based studies have used different Brazilian surveys for research, contributing to an increase in the body of evidence on fruit and vegetable consumption. For example, using data from Vigitel, the fruit and vegetable consumption trend was analyzed in the same population from 2008 to 2016 and an increase in the prevalence of these indicators was identified ^8^. Another study compared fruit and vegetable consumption for the entire adult population in 2013 and 2019 using data from the *Brazilian National Health Survey*. This study found little difference in overall fruit and vegetable consumption between those years, with a slight increase in fruit consumption but not in vegetable consumption ^19^. And data from the *Brazilian Household Budget Survey* (POF, acronym in Portuguese) showed an increase in the availability of fruit and vegetable in Brazilian households during the 2002-2003, 2008-2009, and 2017-2018 periods, driven primarily by an increase in fruit availability ^20^. Although these studies have different methodologies, scopes, and periods of analysis, they similarly indicate insufficient and unequal consumption among populations. Our results add to this evidence, uncovering the population’s updated consumption trends. We found an increase in fruit and vegetable consumption essentially from 2008 to 2014 but a reduction in the prevalence after 2015, confirming the initial hypothesis that the political, economic, and sanitary crisis in the country since 2015 could affect fruit and vegetable consumption.

Several initiatives by the Brazilian government that aim to address NCD have been implemented, including national plans with goals that encompass promoting fruit and vegetable consumption ^1^. The *Strategic Action Plan to Tackle Noncommunicable Chronic Diseases in Brazil, 2011-2022* was the first action plan of the country on this topic, proposing a 10% increase in the prevalence of recommended fruit and vegetable consumption among Brazilians ^21^. The goal was to achieve a 21.5% prevalence by 2022, but this target was already surpassed in 2011 (22%) ^22^. The second and most current action plan, the *Strategic Action Plan to Tackle Noncommunicable Chronic Diseases (NCD) in Brazil, 2021-2030*, proposes a 30% increase in recommended fruit and vegetable consumption by 2030 ^23^. However, as observed in this study, the reduction trend in the recommended fruit and vegetable consumption will likely hinder meeting this goal.

Such events may be associated with structural changes in Brazil during the investigated periods. The increase in fruit and vegetable consumption in the initial period followed a remarkable period of economic, social, and health policies in the country ^1^ ^,^ ^10^ ^,^ ^11^ ^,^ ^12^ ^,^ ^24^ ^,^ ^25^ ^,^ ^26^. The population adhered to healthier behaviors, including fruit and vegetable consumption, as well as greater engagement in physical activity and a reduction in smoking rates ^27^. Notably, actions during this time included the presence of the Brazilian National Food and Nutritional Security Council (CONSEA, acronym in Portuguese), a presidential advisory council with an important role in promoting the agenda of family farming ^24^ ^,^ ^28^, particularly via initiatives like the Food Acquisition Program (PAA, acronym in Portuguese). This program facilitated the purchase of products from family farmers and distributed them to those facing food insecurity ^10^. Income increases, associated with initiatives like the Brazilian Family Income Program [Programa Bolsa Família] ^12^, played a decisive role in reducing food insecurity among beneficiary families, with a positive impact on food consumption ^29^. Additionally, the expansion of PHC, along with the inclusion of nutritionists in these settings ^25^, facilitated nutritional attention and multidisciplinary care for individuals ^11^. Moreover, the reformulation of the *Dietary Guidelines for the Brazilian Population* ^1^ positioned it as a key tool in food and nutrition actions, especially in the PHC ^26^.

However, recent years have been characterized by political instability and the worsening of the economic crisis ^13^, exacerbated by fiscal adjustments that lowered State expenditures, especially in health and social sectors ^30^, since its implementation in 2016 ^31^. This has been compounded by a reduction in social participation in the formulation and monitoring of food and nutrition policies ^28^ ^,^ ^29^, as well as in important health programs in the PHC ^32^. Considering that the purchase of natural and minimally processed foods typically entails a higher financial cost, families still face obstacles in accessing them during this period, mainly for lower income populations ^33^.

Since 2023, some of the programs and actions aforementioned are once again returning to the political agenda, however no significant changes have yet been observed. The reduction in fruit and vegetable consumption among the same groups reinforces the stagnation in overcoming social differences. This study, along with many others, contributes to the growing body of evidence on entrenched disparities in demand for and use of health services related to lifestyle and illness. Studies conducted in developed and developing countries have consistently shown that being a woman ^20^ ^,^ ^34^, younger individual ^8^ ^,^ ^35^, and having higher schooling levels ^6^ ^,^ ^36^ can be considered protective factors.

Women typically presents greater concern about their health and well-being ^34^. This heightened awareness can be attributed to sociocultural norms that emphasize care and self-care among women. They tend to seek more health information and medical advice, leading to greater adoption of health recommendations and lifestyle changes ^37^. The individual’s aging process can often serve as a catalyst for behavior change, as part of recommendations for disease treatment ^38^, while younger individuals tend to have higher ultra-processed food consumption ^35^. A greater consumption of fruit and vegetable among women and older adults was also described in a study carried out with data from the *Behavioral Risk Factor Surveillance System* (BRFSS), the largest telephone survey on NCD carried out in the United States ^9^.

Regarding schooling, individuals with a higher schooling generally tend to prioritize their health ^39^ and consequently adopt healthier behaviors ^6^ ^,^ ^36^. This happens because education contributes to a better understanding of health-related information and an increased awareness of one’s health status. Education can also be seen as a proxy for individual income. This would facilitate access to purchasing these foods ^39^. Despite this, our findings indicate a reduction in the regular and recommended fruit and vegetable consumption in the population, even among those with higher schooling. This suggests that economic changes in the country may have affected individuals with higher incomes as well. Data from the latest POFs (2002-2003, 2008-2009, and 2017-2018) showed an increase in fruit acquisition among the poorest strata, while it remained unchanged among the richest ^20^. Furthermore, the acquisition of ultra-processed foods has grown across all strata over the years ^20^. A recent study highlighted that during the COVID-19 pandemic, ultra-processed foods became cheaper than fresh and minimally processed foods, impacting the purchase of fruit and vegetable, especially among the poorest social classes ^40^.

The decrease in fruit and vegetable consumption represents a deterioration in dietary quality and diversity among the Brazilian population, impacting not only the profile of micronutrients and fiber intake in the diet but also contributing to worsening populations’ health, and a weakening in the food culture of the country ^1 ,2,3^. A set of actions and policies can strategically improve the population’s food intake by discouraging the consumption of ultra-processed foods and encouraging fruit and vegetable consumption. Simple advances have been made, such as changes in food labeling, with an emphasis on front labeling indicating specific critical nutrients present mainly in ultra-processed foods ^41^. Considering the experience of other countries, it is expected that front labeling will enable more informed food choices, consequently reducing ultra-processed food consumption. Furthermore, an indirect effect of healthy food consumption, such as fruit and vegetable, can be expected by shifting consumption. Economic tools, such as the taxation of unhealthy foods concomitant with the subsidy of healthy foods, have been recommended, recognizing that price plays a significant role in food choice ^42^. Reducing the price of healthy foods, such as fruit and vegetable, has been found to be more effective than just increasing the price of unhealthy foods. In fact, even a modest decrease of about 10% in the price of fruit and vegetable can lead to a significant increase of up to 12% in their consumption. On the other hand, a 10% increase in the price of unhealthy foods, such as fast foods, typically results in a modest decrease of about 6% in their consumption ^43^.

Therefore, efforts to increase the direct consumption of fruit and vegetable must be prioritized with strategies that focus on supporting the production, supply, and access to these foods ^21 ,22,23^. A previous study evaluated different intervention scenarios to increase fruit and vegetable consumption in New York City neighborhoods (United States), revealing that two strategies were associated with increased fruit and vegetable consumption: increasing the number of stores in low-income neighborhoods with limited access to healthy foods ^44^ and reducing fruit and vegetable prices through tax subsidies or direct financial incentives ^43^ ^,^ ^44^.

In Brazil, policies aimed at offering low-cost meals and purchasing food from local family farmers to provide school meals have proven to be successful in providing indirect access to healthy foods ^45^ ^,^ ^46^. Additionally, it is crucial to ensure comprehensive care practices for individuals within the scope of PHC and promote adequate and healthy nutrition ^26^. Given the scenario of increasing NCD, promotion actions should target both users and healthcare teams by providing professional training and implementing care protocols that involve multidisciplinary teams ^26^.

We must point out issues and recognize the limitations of the study. Firstly, regarding the inclusion of fruit juices in the calculation of daily fruit consumption. Fruit juices have a historical precedent, with these beverages becoming a staple in many cultures as an alternative to water. Although fruit juices can be nutritious, they have different nutritional properties compared to whole fruits. For these reasons, the international “at least five per day” campaign recommends that juices should account as only one portion per day, regardless of the amount consumed ^47^. This guideline ensures that people benefit from the full spectrum of nutrients and fiber that whole fruit and vegetable provide, promoting overall health and well-being. The World Cancer Research Foundation also recognizes that “*natural fruit juice is a source of healthy nutrients, but it also contains a lot of sugar and loses most of the fiber obtained from eating the whole fruit. Therefore, it is best not to drink more than one glass (150 mL) per day*” ^47^ (p. 151). To ensure the adequacy of the analyses performed, we carried out additional ones excluding juices from the estimates, yielding similar results (data not shown). Furthermore, it is also important to explain the absence of data collection in 2022 and the simplified collection in 2023. In the Vigitel 2023, the report indicates that interviews were concentrated in a few months of the year, there was a reduction in sample size, and interviews via mobile phone were introduced, requiring careful analysis and comparison of estimates ^15^. Likewise, it is important to consider Vigitel’s type of data collection, a telephone survey with self-reported information. While self-reported information is frequently used in health surveys due to ease and low-cost administration in large population samples ^48^, they may produce biased estimations. However, it is plausible to assume that such limitations remain consistent over time, thereby minimizing their impact on the identified trends or annual variations. Regarding Vigitel, a study comparing results from the system with a survey that used a gold standard methodology (household) showed similar outcomes ^49^. Additionally, the Vigitel sample was limited to individuals with at least a landline in Brazilian capitals, an inherent characteristic of the research methodology. However, Vigitel employs appropriate weighting factors to adjust estimates and correct differences between populations with and without a landline. Therefore, it is possible to extrapolate the results to the whole Brazilian population.

## Conclusion

The prevalence of fruit and vegetable consumption showed unfavorable evolution over the 15 editions studied. While there were significant changes in fruit and vegetable consumption in the country, characterized by an increase from 2008 to 2014, there was a substantial reduction in consumption from 2015 to 2023. Men, young adults, and adults with higher schooling levels were the most affected by the reduction in fruit and vegetable consumption. These results highlight the importance of expanding and strengthening public health actions aimed at improving food consumption and conducting continuous surveillance to develop and enhance effective health policies.

## References

[1] Departamento de Atenção Básica. Secretaria de Atenção à Saúde. Ministério da Saúde (2014). Guia alimentar para a população brasileira.

[2] Cena H, Calder PC (2020). Defining a healthy diet evidence for the role of contemporary dietary patterns in health and disease. Nutrients.

[3] Wallace TC, Bailey RL, Blumberg JB, Burton-Freeman B, Chen O, Crowe-White KM (2020). Fruits, vegetables, and health a comprehensive narrative, umbrella review of the science and recommendations for enhanced public policy to improve intake. Crit Rev Food Sci Nutr.

[4] World Health Organization Healthy diet. Update August 2018..

[5] World Health Organization (2003). Diet nutrition and the prevention of chronic diseases. Report of a Joint WHO/FAO Expert Consultation..

[6] Frank SM, Webster J, McKenzie B, Geldsetzer P, Manne-Goehler J, Andall-Brereton G (2019). Consumption of fruits and vegetables among individuals 15 years and older in 28 low-and middle-income countries. J Nutr.

[7] Daniels ME, Donilon TM, Bollyky TJ (2014). The emerging global health crisis. Noncommunicable diseases in low- and middle-income countries..

[8] Silva LES, Claro RM (2019). Tendências temporais do consumo de frutas e hortaliças entre adultos nas capitais brasileiras e Distrito Federal, 2008-2016. Cad Saúde Pública.

[9] Lee-Kwan SH, Moore LV, Blanck HM, Harris DM, Galuska D (2017). Disparities in state-specific adult fruit and vegetable consumption - United States, 2015.. MMWR Morb Mortal Wkly Rep.

[10] Brasil (2003). Lei nº 10.696, de 2 de julho de 2003. Dispõe sobre a repactuação e o alongamento de dívidas oriundas de operação de crédito rural, e dá outras providências.. Diário Oficial da União.

[11] Bortolini GA, Pereira TN, Nilson EAF, Pires ACL, Moratori MF, Ramos MKP (2021). Evolution of nutrition actions in primary health care along the 20-year history of the Brazilian National Food and Nutrition Policy. Cad Saúde Pública.

[12] Brasil (2004). Lei nº 10.836, de 9 de janeiro de 2004. Cria o Programa Bolsa Família e dá outras providências.. Diário Oficial da União.

[13] Instituto Brasileiro de Geografia e Estatística Pesquisa Nacional por Amostra de Domicílios Contínua (PNAD contínua): rendimento de todas as fontes..

[14] Rede Brasileira de Pesquisa em Soberania e Segurança Alimentar e Nutricional Food insecurity and COVID-19 in Brazil..

[15] Ministério da Saúde (2023). Vigitel Brasil 2023: Vigilância de Fatores de Risco e Proteção para Doenças Crônicas por Inquérito Telefônico. Estimativas sobre frequência e distribuição sociodemográfica de fatores de risco e proteção para doenças crônicas nas capitais dos 26 estados brasileiros e no Distrito Federal em 2023.

[16] Ministério da Saúde (2022). Vigitel Brasil 2006-2021: Vigilância de Fatores de Risco e Proteção para Doenças Crônicas por Inquérito Telefônico. Estimativas sobre frequência e distribuição sociodemográfica do estado nutricional e consumo alimentar nas capitais dos 26 estados brasileiros e no Distrito Federal entre 2006 e 2021: estado nutricional e consumo alimentar..

[17] Instituto Brasileiro de Geografia e Estatística (2020). Pesquisa Nacional de Saúde 2019. Percepção do estado de saúde, estilos de vida, doenças crônicas e saúde bucal: Brasil e grandes regiões.

[18] Antunes JLF, Cardoso MRA (2015). Uso da análise de séries temporais em estudos epidemiológicos. Epidemiol Serv Saúde.

[19] Santin F, Gabe KT, Levy RB, Jaime PC (2022). Food consumption markers and associated factors in Brazil distribution and evolution, Brazilian National Health Survey, 2013 and 2019. Cad Saúde Pública.

[20] Levy RB, Andrade GC, Cruz GL, Rauber F, Louzada MLC, Claro RM (2022). Three decades of household food availability according to NOVA - Brazil, 1987-2018. Rev Saúde Pública.

[21] Departamento de Análise de Situação de Saúde. Secretaria de Vigilância em Saúde. Ministério da Saúde (2011). Plano de Ações Estratégicas para o Enfrentamento das Doenças Crônicas não Transmissíveis (DCNT) no Brasil 2011-2022.

[22] Malta DC, Silva AG, Teixeira RA, Machado IE, Coelho MRS, Hartz ZMA (2019). Avaliação do alcance das metas do plano de enfrentamento das doenças crônicas não transmissíveis no Brasil, 2011-2022. An Inst Hig Med Trop (Lisb).

[23] Departamento de Análise em Saúde e Vigilância de Doenças Não Transmissíveis. Secretaria de Vigilância em Saúde. Ministério da Saúde (2021). Plano de Ações Estratégicas para o Enfrentamento das Doenças Crônicas e Agravos não Transmissíveis no Brasil 2021-2030.

[24] Brasil (2006). Lei nº 11.346, de 15 de setembro de 2006. Cria o Sistema Nacional de Segurança Alimentar e Nutricional - SISAN com vistas em assegurar o direito humano à alimentação adequada e dá outras providências.. Diário Oficial da União.

[25] Ministério da Saúde (2008). Portaria nº 154, de 24 de janeiro de 2008. Cria os Núcleos de Apoio à Saúde da Família.. Diário Oficial da União.

[26] Departamento de Atenção Básica. Secretaria de Atenção à Saúde. Ministério da Saúde (2013). Política Nacional de Alimentação e Nutrição.

[27] Silva AG, Teixeira RA, Prates EJS, Malta DC (2021). Monitoring and projection of targets for risk and protection factors for coping with noncommunicable diseases in Brazilian capitals. Ciênc Saúde Colet.

[28] Castro IRR (2019). The dissolution of the Brazilian National Food and Nutritional Security Council and the food and nutrition agenda. Cad Saúde Pública.

[29] Martins APB, Monteiro CA (2016). Impact of the Bolsa Família program on food availability of low-income Brazilian families a quasi experimental study. BMC Public Health.

[30] Menezes APR, Moretti B, Reis AAC (2019). The future of the SUS impacts of neoliberal reforms on public health - austerity versus universality. Saúde Debate.

[31] Brasil (2016). Emenda Constitucional nº 95, de 15 de dezembro de 2016. Altera o Ato das Disposições Constitucionais Transitórias, para instituir o Novo Regime Fiscal, e dá outras providências.. Diário Oficial da União.

[32] Mattos MP, Gutiérrez AC, Campos GWS (2022). Construction of the historical-regulatory standard of the Expanded Family Health Center. Ciênc Saúde Colet.

[33] Maia EG, Passos CM, Levy RB, Martins APB, Mais LA, Claro RM (2020). What to expect from the price of healthy and unhealthy foods over time The case from Brazil. Public Health Nutr.

[34] Silva ZP, Ribeira MCSA, Barata RB, Almeida MF (2011). Perfil sociodemográfico e padrão de utilização dos serviços de saúde do Sistema Único de Saúde (SUS), 2003-2008. Ciênc Saúde Colet.

[35] Costa CS, Sattamini IF, Steele EM, Louzada MLC, Claro RM, Monteiro CA (2021). Consumption of ultra-processed foods and its association with sociodemographic factors in the adult population of the 27 Brazilian state capitals (2019). Rev Saúde Pública.

[36] Barros MBA, Medina LPB, Lima MG, Sousa NFS, Malta DC (2022). Changes in prevalence and in educational inequalities in Brazilian health behaviors between 2013 and 2019. Cad Saúde Pública.

[37] Grzymislawska M, Puck EA, Zawada A, Grzymislawski M (2020). Do nutritional behaviors depend on biological sex and cultural gender. Adv Clin Exp Med.

[38] Barros MBA, Francisco PMSB, Zanchetta LM, César CLG (2011). Tendências das desigualdades sociais e demográficas na prevalência de doenças crônicas no Brasil, PNAD 2003-2008. Ciênc Saúde Colet.

[39] Pampel FC, Krueger PM, Denney JT (2010). Socioeconomic disparities in health behaviors.. Annu Rev Sociol.

[40] Andrade GC, Caldeira TCM, Mais LA, Martins APB, Claro RM (2024). Food prince trends during the COVID-19 pandemic in Brazil. PLoS One.

[41] Agência Nacional de Vigilância Sanitária (2020). Resolução de Diretoria Colegiada nº 429, 8 de outubro de 2020. Dispõe sobre a rotulagem nutricional dos alimentos embalados.. Diário Oficial da União.

[42] World Health Organization 'Best buys' and others recommended interventions for the prevention and control of noncommunicable diseases..

[43] Afshin A, Peñalvo JL, Gobbo LD, Silva J, Michaelson M, O'Flaherty M (2017). The prospective impact of food pricing on improving dietary consumption: a systematic review and meta-analysis.. PLoS One.

[44] Li Y, Zhang D, Thapa JR, Madondo K, Yi S, Fisher E (2017). Assessing the role of access and price on the consumption of fruits and vegetables across New York City using agent-based modeling. Prev Med.

[45] Ministério do Desenvolvimento Social e Combate à Fome Manual do Programa Restaurante Popular..

[46] Brasil (2009). Lei nº 11.947, de 16 de junho de 2009. Dispõe sobre o atendimento da alimentação escolar e do Programa Dinheiro Direto na Escola aos alunos da educação básica; altera as Leis nº 10.880, de 9 de junho de 2004, 11.273, de 6 de fevereiro de 2006, 11.507, de 20 de julho de 2007; revoga dispositivos da Medida Provisória nº 2.178-36, de 24 de agosto de 2001, e a Lei nº 8.913, de 12 de julho de 1994; e dá outras providências.. Diário Oficial da União.

[47] World Cancer Research Foundation American Institute for Cancer Research (2007). Food nutrition, physical activity, and the prevention of cancer: a global perspective.

[48] Riley L, Guthold R, Cowan M, Savin S, Bhatti L, Armstrong T (2016). The World Health Organization STEPwise Approach to Noncommunicable Disease Risk-Factor Surveillance methods, challenges, and opportunities. Am J Public Health.

[49] Caldeira TCM, Soares MM, Silva LES, Veiga IPA, Claro RM (2022). Chronic disease risk and protective behaviors in Brazilian state capitals and the Federal District, according to the National Health Survey and the Chronic Disease Risk and Protective Factors Telephone Survey Surveillance System, 2019.. Epidemiol Serv Saúde.

