# Supplementary material for: Fruit and vegetable consumption among Brazilian adults: trends from 2008 to 2023
Source: Cad Saude Publica. 2025 Feb 7;41(1):e00032424. doi: 10.1590/0102-311XEN032424 (PMC11805522; doi:10.1590/0102-311XEN032424)
Supplement: Supplementary file 1 [file 1678-4464-csp-41-01-EN032424-s.pdf]

SUPPLEMENTARY MATERIAL

Table S1 Percentage of the adult population (≥ 18 years) that consumes fruits and vegetables on five or more days of the week (regular consumption) in Brazilian state capitals and in the Federal District, according to sociodemographic characteristics. *Risk and Protective Factors Surveillance System for Chronic Noncomunicable Diseases Through Telephone Interview* (Vigitel), 2008-2023 (n = 697,549).

**Characteristics**	**2008 (%)**	**2009 (%)**	**2010 (%)**	**2011 (%)**	**2012 (%)**	**2013 (%)**	**2014 (%)**	**2015 (%)**	**2016 (%)**	**2017 (%)**	**2018 (%)**	**2019 (%)**	**2020 (%)**	**2021 (%)**	**2023 (%)**
Sex															
Male	26.4	25.7	26.0	27.5	26.9	29.6	29.4	31.3	28.8	27.8	27.7	27.9	26.2	26.2	27.9
Female	38.6	37.7	37.2	39.0	40.1	41.5	42.5	43.1	40.7	40.4	39.2	39.8	38.2	41.0	35.3
Age (years)															
18-24	24.6	24.3	26.6	24.6	24.8	27.1	27.5	29.3	27.4	26.0	28.2	26.3	23.2	26.3	26.0
25-34	29.6	27.0	27.5	28.4	29.4	30.6	33.9	35.3	31.7	30.5	30.2	31.4	26.5	31.1	28.0
35-44	31.7	33.0	30.0	32.9	33.5	34.5	33.9	35.7	33.4	32.4	32.5	32.7	31.8	31.0	27.5
45-54	37.0	35.1	35.5	36.5	37.7	41.0	38.7	39.2	38.1	38.3	36.3	35.7	35.2	36.2	34.0
55-64	40.7	41.0	40.4	42.9	42.1	44.6	44.6	44.6	42.2	41.7	38.9	40.1	40.5	40.3	37.4
65 and more	45.3	43.2	42.1	48.8	46.2	47.8	47.6	48.1	44.7	45.1	42.1	44.2	45.3	44.7	43.5
Schooling (years of study)															
0-8	29.5	28.6	27.1	30.7	29.7	32.1	32.4	33.0	29.9	31.2	30.5	30.8	29.4	32.9	28.1
9-11	31.0	29.6	30.4	30.5	31.2	33.4	33.4	33.8	32.0	31.1	29.8	29.9	28.3	28.2	28.6
12 and more	43.2	43.1	43.0	43.3	45.0	45.3	46.5	48.9	44.3	41.9	42.1	42.5	40.3	42.4	38.9
**Total**	33.0	32.2	32.0	33.7	34.0	36.0	36.5	37.6	35.2	34.6	33.9	34.3	32.7	34.2	31.9

Table S2 Percentage of the adult population (≥ 18 years) that consumes five or more daily servings of fruits and vegetables on five or more days of the week (recommended consumption), in Brazilian state capitals and in the Federal District, according to sociodemographic characteristics. *Risk and Protective Factors Surveillance System for Chronic Noncomunicable Diseases Through Telephone Interview* (Vigitel), 2008-2023 (n = 697,549).

**Characteristics**	**2008 (%)**	**2009 (%)**	**2010 (%)**	**2011 (%)**	**2012 (%)**	**2013 (%)**	**2014 (%)**	**2015 (%)**	**2016 (%)**	**2017 (%)**	**2018 (%)**	**2019 (%)**	**2020 (%)**	**2021 (%)**	**2023 (%)**
Sex															
Male	15.8	15.8	16.0	17.5	17.6	19.3	19.3	21.0	19.4	18.5	18.4	18.4	17.9	16.9	19.3
Female	23.7	23.9	22.5	25.8	27.2	27.3	28.2	28.9	28.7	28.2	27.2	26.8	26.3	26.4	23.2
Age (years)															
18-24	15.6	15.8	16.9	17.3	17.7	18.9	19.2	21.0	20.7	19.6	20.3	19.0	16.9	18.4	20.2
25-34	18.3	17.3	17.2	19.2	20.4	21.5	22.7	25.3	22.5	21.6	21.6	21.5	18.0	21.8	20.8
35-44	19.4	21.5	18.0	21.0	22.5	22.8	23.4	24.2	23.6	23.9	21.9	22.1	23.2	21.0	18.2
45-54	22.3	21.5	22.0	23.8	24.2	26.2	25.9	26.3	25.9	25.4	24.9	23.9	24.7	22.2	21.5
55-64	23.6	25.1	25.7	27.0	28.5	29.3	28.7	28.8	28.6	27.5	26.6	26.3	27.9	24.8	24.9
65 and more	26.3	25.0	22.2	29.9	28.4	26.8	27.8	27.3	28.2	26.9	25.1	26.6	27.7	25.0	24.8
Schooling (years of study)															
0-8	16.9	16.8	15.3	18.9	18.6	19.4	20.2	20.1	19.7	19.5	19.3	19.0	19.1	17.8	17.1
9-11	19.6	19.0	19.1	20.6	21.2	23.1	22.5	23.2	23.0	22.1	20.9	20.2	19.8	18.7	19.5
12 and more	27.1	28.5	27.4	28.9	31.4	30.1	31.9	34.6	30.8	29.7	29.4	29.5	28.3	29.5	27.2
**Total**	20.0	20.2	19.5	22.0	22.7	23.6	24.1	25.2	24.4	23.7	23.1	22.9	22.5	22.1	21.4
